# Biomarkers of Physical Frailty and Sarcopenia: Coming up to the Place?

**DOI:** 10.3390/ijms21165635

**Published:** 2020-08-06

**Authors:** Anna Picca, Riccardo Calvani, Matteo Cesari, Francesco Landi, Roberto Bernabei, Hélio José Coelho-Júnior, Emanuele Marzetti

**Affiliations:** 1 Fondazione Policlinico Universitario “Agostino Gemelli” IRCCS, 00168 Rome, Italy; anna.picca1@gmail.com (A.P.); francesco.landi@unicatt.it (F.L.); emanuele.marzetti@policlinicogemelli.it (E.M.); 2 Department of Clinical Sciences and Community Health, Università di Milano, 20122 Milan, Italy; matteo.cesari@unimi.it; 3 Geriatric Unit, Fondazione IRCCS Ca’ Granda Ospedale Maggiore Policlinico, 20122 Milan, Italy; 4 Department of Geriatric and Orthopedic Sciences, Università Cattolica del Sacro Cuore, 00168 Rome, Italy; coelhojunior@hotmail.com.br

**Keywords:** cytokines, extracellular vesicles, exosomes, geroscience, gut dysbiosis, inflammation, metabolomics, mitochondrial dysfunction, physical performance, skeletal muscle

## Abstract

Physical frailty and sarcopenia (PF&S) recapitulates all the hallmarks of aging and has become a focus in geroscience. Factors spanning muscle-specific processes (e.g., mitochondrial dysfunction in skeletal myocytes) to systemic changes (e.g., inflammation and amino acid dysmetabolism) have been pinpointed as possible contributors to PF&S pathophysiology. However, the search for PF&S biomarkers allowing the early identification and tracking of the condition over time is ongoing. This is mainly due to the phenotypic heterogeneity of PF&S, its unclear pathophysiology, and the frequent superimposition of other age-related conditions. Hence, presently, the identification of PF&S relies upon clinical, functional, and imaging parameters. The adoption of multi-marker approaches (combined with multivariate modeling) has shown great potential for addressing the complexity of PF&S pathophysiology and identifying candidate biological markers. Well-designed longitudinal studies are necessary for the incorporation of reliable biomarkers into clinical practice and for unveiling novel targets that are amenable to interventions.

## 1. Introduction

Sarcopenia is the progressive decline in muscle mass and strength that occurs during aging [1]. This condition is a hot topic in geriatric research and a public health priority [2], as it exposes older adults to increased risk of negative health-related events (such as disability, loss of independence, institutionalization, and death) [1]. However, its phenotypic heterogeneity, the unclear pathophysiology, and the frequent superimposition of other age-related conditions hamper the study of sarcopenia as a single phenomenon [3]. As a result, an univocal operational definition of sarcopenia is still missing, as are specific biomarkers that could be used, either in clinics or in research [4]. At the clinical level, sarcopenia overlaps with frailty and the age-related decline in physiologic reserve and homeostatic capacity, which predisposes older adults to a wide range of negative health-related events—including falls, morbidity, disability, hospitalization, institutionalization, and mortality [5]. In this scenario, the recognition of physical frailty and sarcopenia (PF&S) as a new entity, and its operationalization in the Sarcopenia and Physical fRailty IN older people: multi-componenT Treatment strategies (SPRINTT) project, have set a remarkable precedent for its clinical and regulatory recognition [6].

The decline in physical function is, indeed, the most evident change that occurs during sarcopenia, and a cardinal criterion for the identification of PF&S [7,8,9,10]. Physical function refers to a construct encompassing simple single-joint (e.g., handgrip strength) and multi-joint complex movements (e.g., walking speed), and may be assessed through a large array of tests [11,12]. Changes in physical performance occur slowly across years [11,13,14,15] and show great heterogeneity among people from different socioeconomic backgrounds [14]. This implies that age- and country-specific cut-off values might be needed in order to identify physical dysfunction [16]. At the same time, when considering physical dysfunction as the ultimate outcome of muscle failure [17], numerous cellular and molecular changes may occur alongside this phenomenon.

When exploring the pathways and processes involved in PF&S pathophysiology, several factors, from muscle-specific mitochondrial dysfunction to systemic changes (e.g., inflammation and amino acid dysmetabolism), have been pinpointed [9,10,18]. It is still unclear whether these processes share common roots and how cell-based alterations spread and are detected at the systemic level, contributing to the disabling cascade that characterizes PF&S. Low-grade inflammation and amino acid dysmetabolism have recently been associated with a specific pattern of small extracellular vesicles (sEVs) of mitochondrial origin, namely mitochondrial-derived vesicles (MDVs), which are proposed to function as shuttles that allow crosstalk between biological systems [7]. However, little is known about their complex regulatory network.

**Figure 1:**
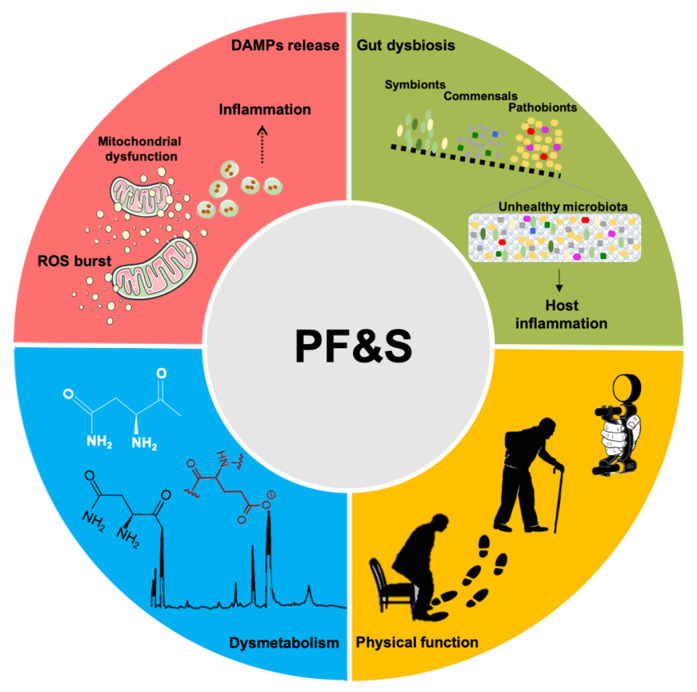
Schematic representation of the main pathophysiological pathways contributing to physical frailty and sarcopenia (PF&S) (i.e., inflammation, gut dysbiosis, declines in physical function, and dysmetabolism), and related biomarkers. Abbreviations: DAMPs, damage-associated molecular patterns; ROS, reactive oxygen species.

A conceptual framework has recently been agreed upon for the selection of blood-borne biomarkers to verify the existence of shared “hallmarks of aging” that can be targeted to extend the health span [19]. The pathophysiology of PF&S recapitulates all the hallmarks of aging [19,20,21], which makes it a prototypical geroscience condition [22]. In this scenario, multivariate analyses of biomediators, pertaining to different domains, may enable the identification of biomarkers for PF&S that capture its pathophysiological complexity (Figure 1) [7,8,9,10,23]. The identified pathways may, eventually, be used for drug development.

Here, we will provide an overview of imaging, functional, and biological markers currently available for PF&S. We will also illustrate the prospect of exploiting specific biological processes to identify new biomarkers for the condition and to develop personalized interventions.

## 2. Imaging and Functional Markers

When Irwin Rosenberg prompted health professionals to pay serious attention to sarcopenia [24], he referred exclusively to age-related muscle atrophy, while changes in physical function and mobility were seen as possible secondary outcomes. Indeed, sarcopenia and muscle atrophy were synonyms until the 2000s [25,26,27], after which findings from observational studies indicated that losses in physical function occurred earlier, progressed more rapidly, and were better predictors of negative health-related events than a decline in muscle mass [13,28]. Indeed, the recognition of declining physical performance as a core feature of muscle aging has prompted the appraisal of sarcopenia as a composite condition that encompasses quantitative (mass) and qualitative (strength/function) muscular domains [29,30].

Body imaging techniques (e.g., dual energy x-ray absorptiometry (DXA), computed tomography (CT), and magnetic resonance imaging (MRI)) are popular tools for the quantification of muscle or lean body mass [31]. However, all of them show notable pitfalls. Indeed, the results of DXA are heavily influenced by body thickness, hydration status, and extracellular fluid accumulation. Furthermore, DXA is unable to measure intramuscular adipose tissue [31]. On the other hand, the large-scale implementation of CT and MRI is hampered by high costs, technological complexity, and space requirements [31]. CT also exposes the test person to non-negligible doses of ionizing radiation.

The creatine (methyl-d3) dilution (D3-creatine) method is a recently developed approach that allows the accurate quantification of whole-body muscle mass [32]. The method relies on the irreversible conversion of creatine to creatinine, and its excretion in urine. The enrichment of urine D3-creatinine allows estimating the total body creatine pool size as a proxy for the whole-body skeletal muscle mass. This method involves the oral administration of stable isotope-labeled creatine (D3-creatine), followed by the collection of a single fasted urine sample 48–96 h later. The sample is assayed for creatine and creatinine (deuterated and unlabeled) via liquid chromatography mass spectrometry (LC-MS) [33,34,35]. By means of an algorithm, this method allows us to calculate the total body creatine pool size and the muscle mass from D3-creatinine enrichment in urine [33,34,35]. Estimates of the total body muscle mass, obtained by this method, show remarkable concordance with the whole-body MRI scans [33,34,35]. Differently from the DXA, the muscle mass quantified by the D3-creatine dilution method is strongly correlated with physical performance, and predicts incidents of falls and functional limitations [36,37,38,39].

Physical function starts declining around the third decade of life, with a steeper decrease beyond the age of 50, which suggests that the initial deflection of physical performance might be an early predictor of sarcopenia [11,13,14,15]. However, physical performance refers to a wide construct involving several components, including (but not limited to) muscle strength, power, and mobility. Therefore, several physical abilities are legitimate functional biomarkers of sarcopenia. The identification of specific components of physical function that, more so than others, predict sarcopenia is particularly challenging, especially because knowledge of this condition has grown considerably in the last two decades, as reflected by changes in its conceptual framework and operational definitions [30,40,41].

Muscle strength refers to the amount of force generated by a dynamic muscle contraction [42,43]. Isometric handgrip strength (IHG) and the five-time sit-to-stand test (5×STS) are two simple, inexpensive and quick tests used to assess upper and lower limb muscle strength, respectively, in different settings, including the community, hospitals, and nursing homes [4,29,30]. Cross-sectional [44,45,46,47,48] and prospective studies [44,47,49,50,51,52] have reported that low IHG is significantly associated with poor physical performance, mobility impairment, and disability. Notable findings by Rantanen et al. [52] reported that men with low IHG during midlife were at higher risk of developing physical dysfunction 25 years later [52]. Although several studies support the hypothesis that IHG might predict physical dysfunction (and, likely, also sarcopenia), there is also evidence indicating that IHG is not associated with lower extremity disability [53]. Therefore, the combination of upper and lower limb muscle strength testing might be necessary for adequately framing sarcopenia. The 5×STS is associated with many physical performance tests [54], progressive disability [53], and has been reported as a predictor of sarcopenia in older Brazilian women [55]. However, larger studies, also including older men, are needed to confirm and expand these initial findings.

Muscle power—the capacity to generate force in a short time interval—declines earlier in life and at a faster rate, while presenting a higher degree of association with some mobility tasks than muscle strength [13,56,57]. For instance, results from the InCHIANTI (Invecchiare in Chianti) study indicated that women aged between 50 and 60 years had a 20–30% reduction of lower limb muscle strength, while muscle power was reduced by ~50% [13]. Lower limb muscle power was also indicated as a better discriminator of poor mobility than upper and lower limb muscle strength [13,57].

In an attempt to empower the discriminatory capacity of physical function as a marker of PF&S, researchers have proposed combinations of physical tests that support the assessment of different physical abilities. The short physical performance battery (SPPB) provides a single score based on a person’s performance in balance tests, gait speed, and 5×STS [58]. In the seminal study by Guralnik et al. [58], the authors observed that people with low SPPB scores were almost five times more likely to develop disability in a short time interval. These findings were confirmed by a recent systematic review and meta-analysis [59] that indicated that lower SPPB was associated with worsening activities of daily living (ADL) and instrumental ADL.

Poor physical function is associated with chronic low-grade inflammation and antioxidant markers in older adults [60,61,62,63,64], thus suggesting that physical dysfunction is likely mediated by cellular mechanisms that, in combination with functional assessment, may be more informative than physical function alone.

## 3. Inflammation-Related Biomarkers

Dysregulation of the cytokine network is a major driver of aging and related conditions [65,66,67,68]. However, the inclusion of inflammatory markers in clinical practice, as a tool for identifying specific conditions, is far from being reached [19]. A core inflammatory profile with gender-specific signatures has been identified in the context of PF&S as composed by higher levels of C-reactive protein and lower concentrations of myeloperoxidase (MPO), interleukin (IL) 8, monocyte chemoattractant protein 1, and platelet-derived growth factor-BB (PDGF-BB). This set of mediators includes markers related to immunosenescence [69,70], micronutrient intake imbalance [71,72], and impaired muscle regeneration, in response to specific stimuli, such as oxidative stress [73,74,75,76,77]. Senescent cells face morphological and functional reshaping, manifested by a senescence-associated secretory phenotype (SASP) [78]. The SASP fingerprint is composed of ILs, chemokines, growth factors, proteases, and extracellular matrix components [78]. The release of these SASP factors induces perturbations in the local microenvironment through autocrine and paracrine signals, in order to prevent proliferation of damaged cells, as well as enabling the recruitment of immune cells and promoting tissue repair [79,80]. Age-related decline in cell quality control systems may induce insufficient clearance of senescent cells and support systemic inflammation, via the overproduction of SASP-related pro-inflammatory cytokines (e.g., IL1β, IL6, and IL8) [81].

A pro-disability effect has long been attributed to the chronic low-grade inflammation observed during aging, referred to as inflamm-aging [68,70,82]. Indeed, higher levels of pro-inflammatory cytokines have been associated with muscle wasting and reduced physical performance [70,83]. This inflammatory response, void of infectious agents and named “sterile inflammation”, is part of the innate immune response triggered by misplaced cellular components [84,85], which are rooted into the “danger theory” of inflammation [86]. According to this theory, the accumulation of damage-associated molecular patterns (DAMPs), and their release from injured cells into the circulation, triggers inflammation via caspase-1 activation and the release of proinflammatory cytokines [87]. Among DAMP molecules, those of mitochondrial origin, in light of their bacterial ancestry, are thought to contribute substantially to inflamm-aging by interacting with Toll-like receptors, NLR family pyrin domain containing 3 inflammasome activation, and cytosolic DNA sensing by the guanosine monophosphate–adenosine monophosphate (GMP–AMP) synthase–stimulator of interferon gene systems [88,89,90].

The mitochondrial involvement in the crosstalk between chronic inflammation and muscle wasting is not surprising given the central role of this organelle in skeletal myocyte viability. Mitochondria are highly interconnected organelles and form a dynamic network that operates via intra-mitochondrial contacts (as well as with the endoplasmic reticulum, lysosomes, and the actin cytoskeleton) to guarantee organelle homeostasis [91,92]. Recently, mitochondrial tubular protrusions, named mitochondrial nanotunnels, have also been identified as additional structures for mitochondrial interconnections, especially in mitochondria immobilized within post-mitotic tissues (e.g., skeletal muscle and cardiac tissue) [92]. These resident organelles are structurally limited in their fusion, and thus may communicate over long distances via nanotunnels [92]. Finally, Golgi-derived vesicles have been identified to participate in mitochondrial dynamics and are newly described contact sites involved in mitochondrial homeostasis [93]. However, the networking ability of mitochondria may be a double-edged sword. Indeed, it is unclear where the boundary lies between the protective role of the inflammation-derived circulating DAMPs and the detrimental effect of over-reactive inflammation. Severe mitochondrial damage and abnormal autophagosome formation were found in the skeletal muscle of IL10-null mice (IL10^tm/tm^), a rodent model of chronic inflammation and frailty [94]. Among mitochondrial DAMPs, circulating mitochondrial DNA (mtDNA) released from damaged organelles is a prominent candidate, linking inflammation with muscle decay [85]. Recent findings by our group support the hypothesis of mitochondrial impairment among the underlying pathogenetic mechanisms of sarcopenia [18]. In particular, as a result of failing quality control systems [91,95,96,97], the release of oxidized cell-free mtDNA and other mitochondrial components within MDVs have been associated with systemic inflammation in older adults with PF&S [7].

The characterization of the composition of exosomes/EVs released by senescent cells (eSASP) has revealed a specific EV SASP signature [98] from which it has been possible to track down their originating cells [99,100]. Therefore, EVs represent a unique tool for capturing the regulatory network of complex conditions and for the identification of cell- and stressor-specific biomarkers. The dissection of these pathways may provide relevant insights into the role played by inflammation in the disabling cascade of PF&S, allowing for the design of personalized treatment strategies.

## 4. Metabolic Markers

The analysis of the wide collection of endogenous metabolites in biological matrices, referred to as metabolomics, allows for the exploration of genotype–phenotype interactions under the impact of the environment. As such, metabolomics supports the analysis of the dynamic changes of organismal function and provides more informative data than gene or protein expression assays [101,102,103,104]. In the context of PF&S, the study of the dynamic metabolic responses to stressors and the characterization of the biochemical pathways involved are particularly relevant, as this condition is tightly associated with metabolic disorders [105,106].

Dietary protein and amino acids are building blocks for muscle protein synthesis and relevant factors for muscle plasticity and trophism [107]. Furthermore, these metabolites are at the crossroads of multiple biological processes, such as inflammation, insulin sensitivity, and redox homeostasis—all of which are candidates for age-related muscle atrophy and dysfunction [108,109]. Therefore, disarrangements in protein–amino acid metabolism may contribute substantially to the pathophysiology of sarcopenia [110,111].

Specific patterns of circulating amino acids have been associated with muscle mass [112] and quality [113] in functionally limited older adults. In particular, lower plasma concentrations of the branched-chain amino acids leucine and isoleucine have been found in sarcopenic, older Norwegian community dwellers [114]. Conversely, higher concentrations of proline characterized older Japanese people with sarcopenia [115]. Finally, it was found that frail older Japanese people had low levels of essential amino acids, compared with their non-frail peers [116]. On coupling the circulating-amino-acid profiling with the multivariate statistical modeling, the discriminatory power of the analytical approach enabled the exploration of the interrelationship between the protein–amino acid dyshomeostasis and PF&S [117]. Indeed, as observed in the BIOmarkers associated with Sarcopenia and PHysical frailty in EldeRly pErsons (BIOSPHERE) study, metabolomics coupled with a partial least squares–discriminant analysis (PLS-DA) allowed the distinct signatures of circulating amino acids and derivatives in older adults, with and without PF&S, to be identified [9,23]. In particular, participants with PF&S were characterized by higher serum levels of asparagine, aspartic acid, citrulline, ethanolamine, glutamic acid, sarcosine, and taurine, while higher concentrations of alpha amino butyric acid and methionine were found in non-PF&S controls [23]. Notably, the metabolic profile of people with PF&S was associated with a decrease in total energy, as well as the quality and quantity of dietary protein intake [23]. In particular, a link between a poor-quality protein diet or (selective) malabsorption and an impaired mitochondrial quality control mechanism was reported [23]. These findings are in keeping with results from the cross-sectional Maastricht Sarcopenia study (MaSS) in which selected nutritional biomarkers (e.g., essential amino acids, branched-chain amino acids, and eicosapentaenoic acid) were decreased in older, sarcopenic adults [118].

In a recent study by our group, multi-marker datasets (which include mediators pertaining to inflammation, metabolism, and mitochondrial dysfunction) were analyzed through an innovative strategy based on Sequential and Orthogonalized Covariance Selection (SO-CovSel) [22]. This approach allowed us to scale down the number of discriminant biomarkers for PF&S to only seven biomolecules (α-aminobutyric acid, asparagine, aspartic acid, citrulline, heat shock protein 72, MPO, and PDGF-BB) [22], which may speed up the implementation of cost-effective biomarkers in the clinical arena. These findings, albeit promising, need to be confirmed in larger-scale, longitudinal studies to integrate composite biochemical measurements into the routine assessment of PF&S.

## 5. Gut Microbiota

Past the age of 70, gut microbiota face substantial compositional and functional modifications [119,120] of which higher inter-individual variability, reduced biodiversity, and colonization by pathobionts are the main features (Figure 1) [119,120]. These changes, collectively referred to as gut dysbiosis, impact host physiology and expose older adults to a higher risk of infections [119,120]. Furthermore, gut dysbiosis is implicated in acute and chronic conditions, beyond the gastrointestinal system [120,121]. The cause–effect inferences between the gut dysbiosis and human diseases cannot yet be established [121]. However, growing evidence supports the existence of a relationship between changes in gut flora, chronic inflammation, and anabolic resistance in muscle wasting [122,123]. As such, gut dysbiosis has been proposed as a factor in the development of PF&S [124,125]. This view is supported by the observation that supplementation with specific *Lactobacillus* strains attenuates muscle wasting in a mouse model of acute leukemia [126], possibly via increased amino acid bioavailability. Indeed, the activity of host- and bacterium-derived proteases and peptidases along the gastrointestinal tract supports the hydrolysis of dietary proteins into peptides and amino acids [127,128]. These bioproducts, in turn, influence microbial growth and survival [129], and regulate energy and the protein homeostasis of the host [130,131]. Amino acids are also precursors of short chain fatty acids (SCFAs), particularly acetate, propionate and butyrate [132], of which acetate is mainly metabolized by muscle cells to produce energy [133].

The age-related decline in the barrier function of the gut mucosa is believed to play a major role in gut dysbiosis [134]. The establishment of a “leaky gut” during aging may favor the entry of gut microbes and/or related products into the circulation, where they trigger inflammation and contribute to immune system dysregulation [135,136]. In physiologic conditions, the gut flora balances pro- and anti-inflammatory responses [137]. Age-associated dysbiosis weakens the action of gut flora against adverse microbial colonization or metabolite removal [138]. In conjunction with this, the secretion of mucins by intestinal cells and the consequent reduction of SCFAs render the intestinal mucosa more permissive of pathogens entering [138,139]. Apart from being an energy source for colonic epithelial cells, SCFAs modulate the release of anti-inflammatory molecules, which are involved in host metabolism and immunity [140]. In particular, butyrate regulates the differentiation of CD4^+^ T cells into regulatory T cells, the induction of transforming growth factors β secretion by epithelial cells, and the production of IL10 and retinoic acid by dendritic cells and macrophages [140]. Therefore, local intestinal inflammation and its propagation, via the leakage of microbes and bacterium-derived inflammatory compounds into the blood, are blunted [140]. In further support of a link between gut dysbiosis and systemic inflammation are findings from a study where young mice were fed a high-fat diet—a known inducer of intestinal permeability [141]. In these rodents, systemic inflammation and leakage of lipopolysaccharide from the intestine into the circulation were described [141].

Chronic inflammation has been indicated as a trait d’union between gut dysbiosis and muscle structural and functional decline [124,125]. The determinants of this crosstalk are unclear, although studies in preclinical models have offered interesting clues. Germ-free mice are preserved from dietary-induced obesity via increased fatty acid metabolism [142]. This phenomenon occurs via activation of the 5′ adenosine monophosphate–activated protein kinase pathway, a gatekeeper of cellular energy status that activates the carnitine:palmitoyl transferase-1 in the muscle. As a result, mitochondria may be fueled by long chain fatty acylCoA and higher levels of the fasting-induced adipocyte factor, linked to the peroxisome proliferator-activated receptor, and gamma coactivator 1-alpha, the master regulator of mitochondrial biogenesis and oxidative metabolism [142]. The orchestration of these activities may assist in counteracting muscle atrophy.

Age-associated changes in the gut flora composition have been related to the progression of diseases and frailty in older adults. Van Tongeren et al. [143] were the first to report an association between the composition of gut microbiota and frailty. In particular, a reduction in the proportion of *Lactobacilli*, *Bacterioides*/*Prevotella*, and *Faecalibacterium prausnitzii*, and an increase in the proportion of *Ruminococcus*, *Atopobium*, and *Enterobacteriacae* were found in individuals showing high frailty scores [143]. The reduced abundance of butyrate-producing bacteria (e.g., *Faecalibacterium prausnitzii*) in frail older adults suggests a positive role for butyrate, namely in reinforcing the tight junctions of intestinal cells and preventing microbial spread into the circulation [144]. The reduced inflammation may, in turn, sustain muscle health [145]. Subsequent results from the ELDERMET study provided further confirmation of this hypothesis and linked butyrate-generating bacteria with functional capacity in older community-dwelling adults [146].

Finally, the application of a multi-marker analytical approach allowed identifying patterns of circulating mediators, which are composed of higher serum concentrations of aspartic acid, lower circulating levels of concentrations of threonine, and the macrophage inflammatory protein 1α (associated with increased abundance of *Oscillospira* and *Ruminococcus* microbial taxa, and decreased abundance of *Barnesiellaceae* and *Christensenellaceae* in older people with PF&S) [8]. The relationship between the abundance of specific intestinal bacteria, metabolic markers, and serum levels of specific inflammatory biomolecules suggests the existence of an additional pathway through which changes in gut microbiota may impinge on PF&S pathophysiology [8].

## 6. Conclusions

To date, the identification of PF&S relies upon clinical, functional, and imaging parameters. However, biological mediators pertaining to different domains (e.g., inflammation, amino acid metabolism, and gut microbiota) have been identified as candidate biomarkers for the condition (Table 1).

Although a fairly large number of metabolic, microbial, and inflammatory biomolecules have been investigated for their association with PF&S, it cannot be excluded that other relevant markers might be obtained through the analysis of a larger set of biomediators. In light of this limitation (and the lack of a “gold standard” biomarker that can reliably predict functional impairment in older adults), the incorporation of these biological markers into clinical practice is yet to be achieved. The adoption of multi-marker approaches combined with multivariate modeling has shown great potential for addressing the complexity of PF&S pathophysiology and unveiling novel targets for interventions. Well-designed longitudinal studies are warranted to accomplish these ambitious tasks.

## Figures and Tables

**Table 1:** Biological markers associated with PF&S operationalized, according to the Sarcopenia and Physical fRailty IN older people: multi-componenT Treatment strategies (SPRINTT) project’s definition.

Biological Domain	Biomarkers
Inflammation	CRP, HSP72, IL1β, IL6, IL8, MCP1, MIP1α, MPO, PDGF-BB
Amino acid metabolism	Asparagine, aspartic acid, citrulline, ethanolamine, glutamic acid, sarcosine, taurine, threonine
Gut microbiota	*Barnesiellaceae, Christensenellaceae, Oscillospira, Ruminococcus*

Abbreviations: CRP, C-reactive protein; HSP72, heat shock protein 72; IL, interleukin; MCP1, monocyte chemoattractant protein 1; MIP1α, macrophage inflammatory protein 1α; MPO, myeloperoxidase; PDGF-BB, platelet-derived growth factor-BB.

## Abbreviations

5 × STS	Five-time sit-to-stand
ADL	Activities of daily living
BIOSPHERE	BIOmarkers associated with Sarcopenia and PHysical frailty in EldeRly pErsons
CRP	C-reactive protein
CT	Computed tomography
DAMPs	Damage-associated molecular patterns
DXA	Dual energy x-ray absorptiometry
HSP72	HSP72, heat shock protein 72
IHG	Isometric handgrip strength
IL	Interleukin
MaSS	Maastricht Sarcopenia study
MCP1	Monocyte chemoattractant protein 1
MDVs	Mitochondrial-derived vesicles
MIP1α	Macrophage inflammatory protein 1α
MPO	Myeloperoxidase
MRI	Magnetic resonance imaging
PDGF-BB	Platelet-derived growth factor-BB
PF&S	Physical frailty and sarcopenia
PLS-DA	Partial Least Squares–Discriminant Analysis
SASP	Senescence-associated secretory phenotype
SCFAs	Short chain fatty acids
SPRINTT	Sarcopenia and Physical fRailty IN older people: multi-componenT Treatment strategies
sEVs	Small extracellular vesicle
SO-CovSel	Sequential and Orthogonalized Covariance Selection
SPPB	Short physical performance battery
